# Effects of resistance training protocols on nonlinear analysis of heart rate variability in metabolic syndrome

**DOI:** 10.1590/1414-431X20187459

**Published:** 2018-06-11

**Authors:** N. Turri-Silva, D.M. Garner, S.H. Moosavi, A.L. Ricci-Vitor, D.G.D. Christofaro, J. Netto, L.M. Vanzella, L.C.M. Vanderlei

**Affiliations:** 1 Departmento de Fisioterapia, Faculdade de Ciências e Tecnologia, Câmpus de Presidente Prudente, Universidade Estadual Paulista, Presidente Prudente, SP, Brasil; 2 Department of Biological and Medical Sciences, Oxford Brookes University, Headington Campus, Oxford, United Kingdom

**Keywords:** Autonomic modulation, Nonlinear dynamics, Metabolic syndrome, Resistance training, Exercise

## Abstract

Despite the various standard non-linear measurements used in autonomic modulation (AM) assessments usually being applied to long time-series, such analyses can sometimes be applied to shorter term series. To overcome this disadvantage, chaotic global methods were formulated by putting together heart rate variability (HRV) linear methods. Chaos provides information about vegetative function control related to cardiovascular risks. Applying this method can be useful to investigate the complexity of the health condition after resistance training protocols, as a therapeutic intervention in AM in metabolic syndrome individuals (MetS). This study aimed to compare the effects of two resistance training programs (conventional *vs* functional) in MetS using nonlinear analysis of AM. MetS subjects (n=50) of both sexes aged 40 to 60 years were randomly divided into two programs; a group of 12 people served as a control group. Both groups performed 30 sessions of training. AM was assessed in the chaos domain by chaotic global techniques. The main results showed that both resistance training, functional and conventional, increased chaos when compared to the control group, respectively, observed by chaotic forward parameter (CFP)1 (13.9±17.9 *vs* 12.8±14.4 *vs* -2.23±7.96; P≤0.05) and CFP3 (15.4±19.8 *vs* 21.9±13.2 *vs* -4.82±11.4; P*≤*0.05). In addition, 30 sessions of both resistance programs increased chaos, and non-linear analysis enabled discrimination of AM after interventions when compared to the control group.

## Introduction

Heart rate variability (HRV) might be applied to assess cardio-autonomic modulation as a simple, reliable, and non-invasive method of monitoring the autonomic nervous system (ANS) (1,2). There are several ways to evaluate the ANS (3). In general, linear analysis cannot calculate the extent of complexity in disease states by time or frequency domain (3). On the other hand, non-linear measurements have been used to clarify data complexity, being much closer to the human biodynamic system, which behaves in a non-linear way (4). Although some non-linear measurements used in autonomic modulation (AM) analysis can be suitable using short-term series, they usually depend on long-term series of data, as was the case of Shannon Entropy (5) and correlation dimension (6). Although there are many methods in chaos domain (3), other methods can help to comprehend the complex field of HRV. In this context, chaotic global methods were formulated by Garner and Ling (7), who proposed a robust method of chaos analysis to investigate disease conditions and evolution of therapeutic interventions.

Chaotic global methods present chaos domain indexes implemented in groups through different algorithms instead of analyzing each one separately, which possibly increases robustness. Spectral multi-taper method (sMTM), spectral detrended fluctuation analysis (sDFA), and spectral entropy are the basis of the method. The last two apply the standard algorithm to a Welch power spectrum, overcoming the disadvantage of limited-time series, misplacing only the phase information. This method has already been used in metabolic disturbances such as children and youth obesity (8,9) and diabetes (10) demonstrating its effectiveness for the detection of chaos property in time series of HRV. However, this method has not been applied to post-exercise interventions in disease states, as in individuals with metabolic syndrome (MetS), which is a condition that promotes autonomic dysfunction and increases the risk of cardiovascular diseases (1,11).

Raimundo et al. (1) reported parasympathetic and global reduction in MetS. Recently, a systematic review study also indicated that MetS is characterized by autonomic global reduction (11). Both studies reinforce the necessity of intervention studies focusing on HRV improvements in MetS. Additionally, the possibility of MetS complications due to its own risk factors such as hypertension, obesity, high triglycerides, low HDL or/and diabetes (12), and impairment of autonomic function (13,14) strongly increase the risk of cardiovascular diseases. Therefore, improving the knowledge about treatment approach for MetS seems to be necessary.

The effects of exercise training interventions assessed through HRV have been explored previously (15). Among training modalities, resistance training is highlighted due to an inverse association between muscular fitness and strength and the incidence of MetS (16). It is known that people who practice resistance training have 34% lower chance than controls of developing the syndrome (16). Moreover, there is no consensus about the effect of resistance training on resting autonomic modulation in individuals with autonomic dysfunction (14).

There are two types of resistance training: conventional resistance training (CRT) and functional resistance training (FRT). CRT is characterized by local strength stimulus, whereas FRT emphasizes multiple joint and muscle activities simultaneously, combining upper and lower body movements and greater use of the body in every movement to sustain different postures (17–19). Both are commonplace in the world fitness framework and their applicability is appropriate in healthy individuals or as a treatment for cardiovascular diseases (19–21).

Analyzing the effects of resistance training on autonomic modulation through chaos domain can be suitable to better understand the patient’s health status and help the treatment process. Thus, this study analyzed and compared the effects of two resistance training programs (CRT *vs* FRT) on autonomic modulation of MetS patients by the *modus operandi* of chaotic global methods to access the chaotic domains.

## Material and Methods

### Trial design and population

This was an experimental partially randomized controlled clinical trial. This study included male and female volunteers, age-matched between 40 and 60 years who had MetS, were referred by physicians, and classified by the first Brazilian guideline of MetS diagnosis and treatment (I-DBSM) (22). The volunteers were divided into 3 groups (two randomized physically active groups and one non-randomized control group).

The exclusion criteria were physically active volunteers, those who had undertaken any physical training or completed any muscle training at a gym or equivalent in the last 6 months, respiratory diseases, women with amenorrhea, inflammatory or infection symptoms, and individuals with any muscle, tendon, or osteoarticular injuries in any part of the body.

The subjects were informed about the procedures and the study objectives and signed an informed consent form that remained confidential. All procedures were approved by Ethics Committee in Research of Universidade do Estado de Sao Paulo (UNESP) (Process number - CAAE: 17378813.0.0000.5402).

### Experimental design

Data collection was completed at Faculdade de Ciencias e Tecnologia, UNESP and the exercise training was undertaken at a clinic in Presidente Prudente, Brazil. Before physical training, the volunteers were identified, and anthropometric data were collected (body mass and height). The body mass index (BMI) and cardiovascular parameters such as blood pressure and heart rate were collected. The intervals between consecutive heartbeats (RR interval series) were collected by RS800 Polar (Polar Electro OY, Finland), during 30 min in a supine position, always in the morning. In addition, another day was scheduled for the strength test, which was undertaken to determine the individual load to be used in the protocol (the maximum load of each muscle group to be evaluated – called maximum repetition – MR).

All procedures were undertaken twice: baseline and after 30 training sessions (approximately 3 months after starting interventions). The training programs occurred three times per week, totaling 30 training sessions for all volunteers. Two recovery weeks were allowed to emphasize strength gains through recuperative rest (4^th^ and 8^th^ weeks).

The control group remained sedentary overtime; they did not practice any exercise interventions and they were instructed to avoid any kind of physical training activity during the protocol period (supervised or unsupervised physical activities). The total duration of the experimental protocol was from January 2014 until October 2015 due to different time frames to find volunteers according to the physicians' referral. Thus, the training protocol was completed by periods, and 6 individuals were considered enough to initiate the protocol.

### Experimental groups and randomizations

The study groups were: FRT, functional resistance training (machines and complex moving levers); CRT, conventional resistance training (machines); CG, control group (no exercise). The FRT and CRT groups underwent the training protocols, while CG did not practice any physical activity during the process. The control group was composed by volunteers who refused to participate in any intervention protocol.

The volunteers for training protocols were recruited over a period of time. Therefore, the randomized training groups were composed of at least six and a maximum of twelve individuals per training period. The randomization process was achieved by a draw, with the number of tickets equally distributed between FRT and CRT when the sample size was even. When the sample size was odd, the subsequent draw included an extra ticket for the group that had with fewer volunteers in the previous draw. The random allocation sequence, the participant's enrollment, and the assignment of participants to interventions were determined by two trained physiotherapist researchers.

### Training programs

Before starting the training protocol, the groups were familiarized with the equipment and volunteers had their strength evaluated by their MR. From the beginning of the training, the loads increased progressively until the last session of training was completed. The recovery intervals took between 40 to 90 s, depending on the loads. Table 1 describes the dynamic distribution of the loads, the exercise programs, and the training sessions for both training groups (FRT and CRT).

**Table 1.** Training sessions and exercise dynamics.

Weeks	Session	Work volume dynamics (series and repetitions by exercise)	Effort intensity dynamics (exercise load)
1^st^	1 / 2 / 3	2 series × 12 repetitions	30–40% - 1 MR
2^nd^	4 / 5 / 6	2 series × 16 repetitions	30–40% - 1 MR
3^rd^	7 / 8 / 9	2 series × 20 repetitions	30–40% - 1 MR
4^th^		Recovery week	Recovery week
5^th^	10 / 11 / 12	1 series × 16 repetitions	40% - 1 MR
		1 series × 12 repetitions	50% - 1 MR
		1 series × 9 repetitions	60% - 1 MR
6^th^	13 / 14 / 15	1 series × 12 repetitions	50% - 1 MR
		1 series × 9 repetitions	60% - 1 MR
		1 series × 6 repetitions	70% - 1 MR
7^th^	16 / 17 / 18	1 series × 10 repetitions	60% - 1 MR
		1 series × 8 repetitions	70% - 1 MR
		1 series × 6 repetitions	80% - 1 MR
8^th^	19 / 20 /21	1 series × 8 repetitions	70% - 1 MR
		1 series × 6 repetitions	80% - 1 MR
		1 series × 4 repetitions	90% - 1 MR
9^th^		Recovery week	Recovery week
10^th^	22 / 23 / 24	1 series × 6repetitions	80% - 1 MR
		1 series × 4 repetitions	90% - 1 MR
		1 series × 2 repetitions	100% - 1 MR
		1 series × 4 repetitions	90% - 1 MR
		1series × 6 repetitions	80% - 1 MR
11^th^	25 / 26 / 27	1 series × 6 repetitions	80% - 1 MR
		1 series × 4 repetitions	90% - 1 MR
		1 series × 2 repetitions	100% - 1 MR
		1 series × 2 repetitions	100% - 1 MR
		1 series × 4 repetitions	90% - 1 MR
		1 series × 6 repetitions	80% - 1 MR
12^th^	28 / 29 / 30	1 series × 6 repetitions	80% - 1 MR
		1 series × 4 repetitions	90% - 1 MR
		1 series × 2 repetitions	100% - 1 MR
		1 series × 2 repetitions	100% - 1 MR
		1 series × 2 repetitions	100% - 1 MR
		1 series × 4 repetitions	90% - 1 MR
		1 series × 6 repetitions	80% - 1 MR

1 MR: one maximum repetition

The leg exercises were identical for both training groups (leg press, leg curl machine, and extensor machine); however, different exercises for upper limbs were adopted according to each training group, following specific postures. Postures requiring multiple joint and muscular activity simultaneously were adopted for subjects of the FRT group when executing exercises. Dorsal or ventral position in a bench of 45° degrees of inclination was adopted to use crossover machine, being dorsal position for back and pectorals exercises and ventral position for biceps and triceps exercises. These postures were chosen to stimulate contractions of different muscle groups, in addition to specific muscle activity (biceps, triceps, pectoral, and back). For shoulder exercises, the Bozu equipment was utilized to promote instability along the exercise execution in FRT. For CRT, classical executions were adopted for the same muscular groups in specific machines (biceps, triceps, pectoral, and back). All participants completed all training sessions. In case the participant could not attend the training, the missed session was replaced in up to 72 h.

### Subject's security

The training was undertaken at a clinic of Presidente Prudente, São Paulo State, by trained physiotherapists with supervision of a cardiology specialist. Any outstanding health issues were forwarded to the patient’s regular physician. Before, during, and after the sessions, blood pressure and heart rate were monitored and clinical signs such as excessive tiredness, intense sweating, paleness, palpitations, and chest pain were investigated. There were no medical complications during the training sessions. These procedures were sufficient to monitor training intensities and ensure safety.

### Cardiovascular parameters

Blood pressure (BP) was evaluated using a stethoscope and aneroid sphygmomanometer (Welch Allyn Durashock, USA) after at least 15 min of rest, followed by three measurements with a 1-min interval between them and considering the mean value, according to the recommendations from the American Heart Association (23). Heart rate was evaluated using heart monitor (Polar Electro Oy, RS800, Finland).

### Anthropometric evaluation

Height was evaluated in orthostatic position by a stadiometer (Sanny, Brazil) and the body mass by a digital scale (Tanita BC554, Iron Man/Inner Scaner, Germany). Both measures were used to calculate the BMI (body mass/height^2^) of the volunteers.

### Muscle strength evaluations

The muscle strength (in kilograms) was evaluated by an MR test, which evaluates the maximum load in one full cycle of the selected exercise movement (24) using good posture. This step was necessary to determine the correct load to be applied during the training protocol. Before the test, the volunteers undertook a warm-up session (6 to 10 repetitions) with 50% of the load for the first attempt of 1MR. After two min of rest the test was started. The individuals were instructed to complete two repetitions. If both were completed in the first attempt, the second session was performed with a higher load. If the volunteer could not perform a repetition, a second try was indicated, after three to five min of recovery. The load increments occurred according to the subject's perception, until the volunteer’s maximum load without mechanical failure. Tests with more than five attempts were discarded (25). All evaluations for each muscle group occurred before and after the training protocol.

### Heart rate variability

The beat-to-beat heart rate data to analyze the HRV was undertaken in an artificially acclimatized room (21 to 23°C) with relative humidity of 40 to 60%. The data was collected between 07:00 and 12:00 am to minimize interference of circadian rhythms. Individuals were evaluated at rest and awake. Movements and distractions around the room were minimized during the data collection to reduce the anxiety of the volunteers.

The subjects were instructed to refrain from ingesting ANS stimulants such as alcoholic beverages, caffeine, and chocolate for 24 h prior to performing the assessment. The data was collected individually, and the subjects were instructed to remain at rest and avoid conversation during 30 min with spontaneous breathing and in a supine position. The Polar RS800 HR monitor (26) was used for HRV acquisition. Microsoft Excel (Microsoft Office, USA) and Matlab software (Mathworks, USA) were used for the analysis of HRV.

The datasets obtained from the HR monitor were transferred to a computer through the Polar Pro Trainer software, version 5.0 (Polar Electro OY). During data analysis, sections from the RR-intervals were selected. There were 800 consecutive RR intervals containing greater than 95% sinus beats. Subsequently, the data sections were subjected to filtering, firstly by Polar Pro Trainer software and then supplemented by manual inspection with Microsoft Excel software, deleting any interference that could influence the outcome. After these procedures, the txt data files were analyzed by chaotic global techniques (8) regarding specific algorithms on Matlab software.

### Chaotic global method

The chaotic global methods have an index from chaos domain implemented in groups through different algorithms. DFA and Shannon Entropy are present in the method, however in a spectral mode (sDFA and sEntropy, respectively). Furthermore, sMTM is also present, which applies the responsive and adaptive MTM to the data, representing the area between the MTM spectrum and the baseline. It is important to highlight that spectral entropy and sDFA applied to multi-taper spectra, which do not have fixed windows but are adaptive and sensitive, provide results that may have greater chaotic parametric response. Thus, these parameters become high spectral entropy (hs Entropy) and high spectral detrended fluctuation analysis (hsDFA) (27). In this study, the parameters for MTM were: i) sampling frequency of 1Hz; ii) time bandwidth for the discrete prolate spheroidal sequence is 3; iii) fast Fourier transform length of 256; and iv) Thomson's adaptive nonlinear combination method to combine individual spectral estimates.

High spectral entropy (hsEntropy) (27) is a function of the irregularity of amplitude and frequency of the power spectrums peaks. It is derived by applying Shannon entropy to the MTM power spectrum. This output is then normalized so that the sum of the magnitude is equal to unity, giving a normalized power spectrum. We then calculate an intermediate parameter, which is the median Shannon entropy of the value obtained from three different power spectra using the MTM power spectra under three test conditions: a perfect sine wave, uniformly distributed random variables, and finally the experimental oscillating signal. These values are then again normalized mathematically so that the sine wave gives a value of zero, uniformly random variables give unity, and the experimental signal between zero and unity. This final value corresponds to hsEntropy.

hsDFA (28) is determined by calculating the spectral adaptation in the same way as for hsEntropy using a MTM power spectrum with the same settings, but DFA rather than Shannon entropy is the algorithm applied. Importantly, hsDFA is the same as sDFA except the power spectrum is the MTM type rather than that of Welch's.

sMTM (7) is based on the increased intensity of broadband noise in power spectra generated by irregular and chaotic signals. sMTM is the area between the MTM power spectrum and the baseline.

### Chaotic forward parameters (CFP)

There are seven different combinations of three chaotic global parameters, which can be consulted in a previous paper (10). Since hsDFA responds to chaos in the opposite way to the others, we subtracted its value from the unity. All three chaotic global values have equal weighting. It was expected that the CFP, which applies all three values, should be the most robust since it takes the most information and processes in three different ways.

### Normalization and statistical analysis

Descriptive analysis was used to illustrate the baseline characteristics. The normality of the data was analyzed by the Shapiro Wilk test (28). Considering different distributions among the index, parametric and non-parametric tests were applied and reported in the tables. The comparisons between baseline and post-interventions or control group were obtained using the paired Student’s *t*-test (parametric data) or Wilcoxon test (non-parametric data), depending on the data distribution. Data are reported as means±SD or median and interquartile range. The comparisons among three groups were calculated using the relative variation between before and after training, using the analysis of covariance (ANCOVA) adjusted by sex. The Bonferroni post-hoc test was used to detect differences. The effect size of the differences between groups was measured by ETA-squared and considered small if ≥0.01 and <0.06, medium if ≥0.06 and <0.14, and large if ≥0.14 (29). Significance level was set at P<0.05 for all analyses.

## Results

**Figure 1.**
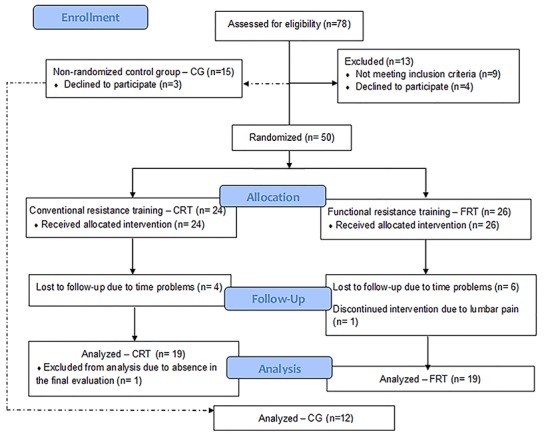
Adapted CONSORT flow diagram for metabolic syndrome individuals following conventional resistance training group (CRT), functional resistance training group (FRT) or control group (CG).

Figure 1 illustrates the distribution of the volunteers adapted from CONSORT recommendations for randomized clinical trials. Considering the number of subjects analyzed in this study, we ensured a test power over 80%, with a significance level of 5% and a two-tailed hypothesis test calculated using the CFP3 index, which presents higher standard deviation considering the relative difference detected.

As indicated in Table 2, except for DBP, there were no significant differences for age, anthropometric data, or cardiovascular parameters. Higher values of DBP were found for FRT compared to CG. However, in general, the results showed homogeneity among the groups, an elementary step before making post-intervention comparisons.

**Table 2:** Baseline characteristics among metabolic syndrome individuals regarding functional resistance training (FRT), conventional resistance training (CRT), and control groups (CG).

Parameters	FRT (n=19)	CRT (n=19)	CG (n=12)
Age (years)	52.32 ± 6.57 (49.15–55.48)	51.42 ± 5.22 (48.91–53.94)	51.21 ± 7.33 (46.98–55.45)
Male	13 (68.42%)	10 (52.63%)	8 (66.67%)
Mass (kg)	84.88 ± 13.21 (78.51–91.24)	89.61 ± 14.19 (82.77–96.45)	83.43 ± 19.93 (71.92–94.94)
Height (m)	1.65 ± 0.11 (1.60–1.70)	1.66 ± 0.09 (1.61–170)	1.67 ± 0.10 (1.62–173)
BMI (kg/m^2^)	31.37 ± 4.50 (29.21–33.54)	32.74 ± 5.10 (30.29–35.19)	29.42 ± 5.26 (26.39–32.46)
HR (bpm)	72.74 ± 10.96 (67.46–71.88)	67.68 ± 8.70 (63.49–71.88)	65.21 ± 8.10 (60.54–69.89)
SBP (mmHg)	129.21 ±19.02 (120.04–138.40)	119.61 ± 12.65 (113.51–125.70)	116.79 ± 18.46 (106.13–127.44)
DBP (mmHg)	85.26 ± 11.48* (79.73–90.80)	77.37 ± 10.05 (72.52–82.21)	75.71 ± 12.07 (68.75–82.68)

Data are reported as means±SD (95%CI). BMI: body mass index; SBP: systolic blood pressure; DBP: diastolic blood pressure; HR: heart rate. *P<0.05 compared to CG (one-way ANOVA followed by Tukey’s test).

About autonomic complexity, Supplementary Table S1 illustrates the comparison of chaotic global index of HRV among the three groups. In relation to the comparisons between pre- and post-intervention or control group, both intervention groups increased chaos for some of the CFP. The conventional group presented significantly higher values than the functional group. However, in general, both groups had higher values post-intervention. There was no significant difference between pre- and post-moments for control group.

Concerning relative differences between groups, higher CFP1 and CFP3 values were observed in FRT and CRT compared to CG (P<0.05). Differences were observed for CFP5 between CRT and CG (P<0.05) and for CFP7 between CRT and FRT (P<0.05). According to the effect size, we found a high magnitude of the effect for CFP1, CFP3, and CFP5 while the other indexes presented low or medium effect. No difference was observed among the groups for CFP2, CFP4, and CFP6 relative values (P>0.05).

## Discussion

The main breakthrough of our study is that structured resistance training improved cardiac autonomic modulation, increasing the chaos and complexity of ANS in MetS individuals and it was independent of the training protocol applied, conventional or functional. We highlight that this finding was observed using nonlinear HRV analysis, which is closer to human biodynamic behavior that works in a non-linear way.

The chaos analysis was made by a comparison with a noisy harmonic oscillation, considering the noise of the signal, which is the opposite of linear methods. Linear methods interpret all regular structure in a data set and can only lead to periodically oscillating solutions, where all irregular behavior is attributed to some random external input to the system (30). Thus, the chaos analysis helps to transpose this limitation.

Considering that the most powerful chaotic global parameter is CFP1, which has the most influence on chaotic outcome when tested by principal component analysis, we could clearly demonstrate the increment of chaos in both intervention groups. These results were further elucidated by the comparison of each intervention group with themselves where basically all chaotic global parameters had a visible increase, even if not always statistically significant.

The present study confirmed that 12 weeks of resistance training protocol can be a reliable intervention for autonomic dynamical balance, increasing the chaos properties in MetS individuals. The resistance exercise can promote other good cardiovascular adaptations already reported in the literature, such as left ventricular hypertrophy (31) and vascular resistance reduction (32) that together can reduce the effort of the heart.

This increase of chaotic response is necessary because it is related to better prognostic and adaptability of the physiological system (33), preparing not just the cardiovascular system but all the underlying systems (vegetative systems) to respond earlier when presented with a new requirement.

Additionally, according to relative differences and the effect size, we found a high magnitude of the effect with significant differences for CFP1, CFP3, and CFP5. As mentioned above, although CFP5 presented a high magnitude of the effect, CFP1 is the most powerful of the chaotic global parameters, and CFP3 along with the first can be considered powerful. CFP1 includes three different chaotic indexes in the same algorithm, which can explain the results found in this study.

The results of this study are particularly important to clarify the autonomic repercussion after functional training program, which has been inserted in the world fitness framework and has been suggested as an improved alternative to the traditional resistance program to improve muscle strength, endurance, coordination, and balance (34). In the autonomic field, there are few studies focusing on this aspect. Recently, Barbosa et al. (19) showed that functional training for 30 sessions with recovering intervals of 24–72 h between sessions could generate improvements in autonomic modulation when analyzed by linear indices of HRV in young healthy individuals.

In the scientific community, it is crucial to test new therapeutic approaches and find scientific evidence before applying them in patients. Thus, the present paper aimed to assist professionals about the autonomic repercussion of the functional training, confirming that there is no significant difference between functional and conventional training in relation to the chaos autonomic modulation for MetS individuals indicating that both methods are good options.

Furthermore, this study overcomes the limitations of other chaotic parameters, which were recently explored by Sassi et al. (30), emphasizing there are possible explanations for the inconclusive findings, including the sensitivity of investigated measures, specifically Lyapunov exponents (35) and correlation dimensions, D_2_ (5), to the limited length and noise of RR series.

Yet, technically oriented articles report new HRV analysis technologies, while medically oriented publications deal with HRV assessment in different physiological and clinical conditions. Unfortunately, there is a noticeable detachment between these two categories. Biomedical engineering literature includes many methods that have never been successfully applied to clinical data. Practical values of advanced novel HRV technologies might thus be difficult to judge (30). This study explored new autonomic tool evaluations, called chaotic global methods, in relation to physiological aspects.

Considering the increasing support for the application of resistance training in individuals with chronic diseases (14,36) this study contributed directly to the knowledge in this field, exploring a therapeutic approach for MetS individuals and a complementary biomarker for treatment evolution, and assisting the performance of health professionals. Furthermore, because this paper provides a practical applicability, we believe researchers and clinics can engage with chaotic data, considering the chaotic global method even with relatively small datasets (800 heartbeats).

As a limitation factor of this study, the control group was a convenience sample and not sex-matched. However, to overcome this aspect, we ran the analysis adjusting for sex. Another limitation is that we did not apply a quantitative method to evaluate physical activities. However, the volunteers were asked about the practice of any physical activities, supervised or unsupervised, and absences were reported. They were also instructed to avoid other physical training activity during the protocol period.

In summary, we found no difference between the effects of training protocols, conventional or functional, on autonomic modulation accessed by the chaotic index in MetS individuals from 40 to 60 years of age. However, strength training was shown to significantly increase complexity of HRV, and is therefore physiologically beneficial. The chaotic global method could identify a complexity increase on autonomic modulation through resistance training.

## Supplementary Material

Click here to view [pdf].
